# Adsorptive Removal of Rhodamine B Using Novel Adsorbent-Based Surfactant-Modified Alpha Alumina Nanoparticles

**DOI:** 10.1155/2020/6676320

**Published:** 2020-12-09

**Authors:** Thi Hai Yen Doan, Thi Phuong Minh Chu, Thi Diu Dinh, Thi Hang Nguyen, Thi Cam Tu Vo, Nhat Minh Nguyen, Bao Huy Nguyen, The An Nguyen, Tien Duc Pham

**Affiliations:** ^1^Faculty of Chemistry, University of Science, Vietnam National University, Hanoi – 19 Le Thanh Tong, Hoan Kiem, Hanoi 100000, Vietnam; ^2^Department of Infrastructure and Urban Environmental Engineering, Hanoi Architectural University, Nguyen Trai, Thanh Xuan, Hanoi 100000, Vietnam; ^3^HUS High School for Gifted Students, University of Science, Vietnam National University, Hanoi, 182 Luong the Vinh, Thanh Xuan, Hanoi 100000, Vietnam; ^4^Marie Curie School, Tran van Lai, My Dinh 1, Nam Tu Liem, Hanoi 100000, Vietnam; ^5^499 Tran Khat Chan, Hai Ba Trung, Hanoi 100000, Vietnam

## Abstract

The objective of the present study is to investigate removal of cationic dye, rhodamine B (RhB), in water environment using a high-performance absorbent based on metal oxide nanomaterials toward green chemistry. The adsorption of sodium dodecyl sulfate (SDS) onto synthesized alpha alumina (*α*-Al_2_O_3_) material (*M*0) at different ionic strengths under low pH was studied to fabricate a new adsorbent as SDS-modified *α*-Al_2_O_3_ material (*M*1). The RhB removal using *M*1 was much higher than *M*0 under the same experimental conditions. The optimal conditions for RhB removal using *M*1 were found to be contact time 30 min, pH 4, and adsorbent dosage 5 mg/mL. The maximum RhB removal using *M*1 achieved 100%, and adsorption amount reached 52.0 mg/g. Adsorption isotherms of RhB onto *M*1 were well fitted by the two-step adsorption model. The electrostatic attraction between positive RhB molecules and negatively charged *M*1 surface controlled the adsorption that was evaluated by the surface charge change with zeta potential and adsorption isotherms. Very high RhB removal of greater than 98% after four regenerations of *M*1 and the maximum removal for all actual textile wastewater samples demonstrate that SDS-modified nano *α*-Al_2_O_3_ is a high-performance and reusable material for RhB removal from wastewater.

## 1. Introduction

Rhodamine B (RhB) has been commonly used as dyes in the industries such as the printings, textiles, papermaking, paints, and leathers [1–3]. A substantial amount of RhB has been released into the environment, polluting the water and causing danger to the biological systems and human life [4–6]. The characteristics of RhB are similar with other synthetic aromatic dyes which are difficultly eliminated out of water due to the high water solubility and difficultly degraded by the light, the temperature, the chemicals, and the microbes [7, 8]. The removal of RhB is important for the wastewater treatment. The conventional techniques basing on the biochemical, physical, and chemical properties are employed to remove the RhB from aqueous solution which are photocatalytic degradation [9, 10], ion exchange [11, 12], membrane filtration [13], and adsorption [6, 14, 15]. However, these techniques show some disadvantages such as the low efficiency, the long consumption time, and the nonbiodegradable product generations [1]. Some researchers studied photocatalytic degradation of RhB from the industrial effluents under the effective factors of the UV radiation, the temperature, the electron acceptor H_2_O_2_, pH [10], and the TiO_2_ dosage [9, 16], introducing the high removal efficiency, the low cost, and the low consumption time. However, the photocatalytic degradation process is more potential to handle RhB from the pretreated wastewater than the raw one [9]. On the other hand, Goto et al. [17] found that the foam separation is one of the most effective methods to remove zwitterionic RhB from solutions in which RhB was adsorbed onto a the bubble surface of the surfactant anionic sodium dodecyl sulfate. Moreover, adsorption is the most suitable methods to remove RhB from aqueous solution [7, 8, 18]. The activated carbons have widely applied as adsorbents to remove RhB due to its simplicity and efficiency [7, 9, 19]. Recently, the activated carbon has been produced from some diversified natural materials such as the orange peels [2], the chestnut peels [20], the resins [12, 14], the almond shells [19], and the palm shells [21]. However, activated carbon is a high-cost material that is not suitable for developing countries [22]. Therefore, many scientists pay more attention in the development of low-cost adsorbents. The RhB can be removed by raw adsorbents or modified-adsorbents. Qin et al. [23] proved that the Fe_3_O_4_/RGO composite is more effective for RhB removal than the activated carbon. The authors also found that the RhB adsorption on the composites was 3.7 times higher of the adsorption capacity and 30 times faster of the adsorption rate than that is on the active carbon [23]. Selvam et al. [24] mentioned that the sodium montmorillonite was the available and cheap clay for eliminating dyes. The removal of RhB from the textile effluents was achieved more highly through the adsorption technique using the purified bentonite clays than the natural one due to the smaller diameter particles and the higher proportion of bentonite in the purified clays [25]. The use of the surfactant-modified-substrates to remove RhB has been proved to be more effective in many studies due to the advantages in modifying the surface properties and the essential surface charge [26, 27]. The adsorption isotherms of surfactants onto the oppositely charged surface fast reach to an equilibrium state that is useful to modify the adsorbent surface [28]. The high efficiency of RhB removal from aqueous solution was found to be 83.0% by the adsorption onto the cationic surfactant-modified-bentonite clay at a high pH of 9.0 [26], or 99.3% RhB removal was achieved by using anionic surfactant-modified-zeolite at a pH of 3 [27]. The adsorption kinetics of RhB using various adsorbents followed a pseudo-second-order model [15, 24, 26, 27]. The adsorption kinetics are strongly depended on the substrate surface and the type of surfactant [28].

In our previous research, RhB was completely removed through the adsorption technique using the anion surfactant sodium dodecyl sulfate (SDS) modified-*γ*-Al_2_O_3_ [29]. Although, *γ*-Al_2_O_3_ has high specific surface area, the *α-*Al_2_O_3_ is the most stable form [30]. Also, *α*-Al_2_O_3_ is the main component of the natural soil so that a comprehensive study on *α*-Al_2_O_3_ is important for further investigation with the real soil. Therefore, the systematically adsorptive removal of RhB using *α*-Al_2_O_3_ was used in the present study. The SDS is applied to modify the *α*-Al_2_O_3_ surface for RhB removal from the wastewater. The adsorption mechanisms are extensively investigated basing on the charging behaviors of adsorbent and adsorption isotherms. The regeneration of adsorbent and application for RhB removal from actual textile wastewater samples are also studied in this work.

## 2. Materials and Methods

### 2.1. Materials

Aluminum nitrate (Al (NO_3_)_3_·9H_2_O) and NaOH pellets, which are analytical reagents, are delivered from Samchun (Korea). Sodium dodecyl sulfate (SDS) (>95% of purify, Wako Pure Chemical Industries, Ltd., Japan) was directly used without further purification as a surface modifier. The critical micelle concentration (CMC) of SDS is measured by the conductometry under different NaCl (p. a, Merck, Germany) concentrations at 22°C mentioned in somewhere [31]. The stock SDS solution of 0.1 M was prepared for adsorption experiments. Rhodamine B (RhB) was purchased from Merck with a molecular weight of 479.02 g/mol, and the purity > 95% is employed as cationic dye. The chemical structures of SDS surfactants and RhB were described elsewhere [29]. The ionic strength was controlled by adding the suitable volume of 0.1 M NaCl. The salt solution was filtered through a 0.2 *μ*m cellulose membrane before using. The pH solution is adjusted by the addition of HCl and NaOH and measuring by a pH meter (Hanna, Woonsocket city, USA). Ultrapure water with a resistance of 18.2 MΩ.cm used in all experiments was daily produced by an ultrapure water system (Labconco, Kansai, MO, USA).

### 2.2. Alpha Alumina Synthesis and Modification

Nanosized alpha alumina (*α*-Al_2_O_3_) was synthesized by the solvothermal method according to our previously published paper [32]. It should be noted that all alumina forms are transformed to *α*-Al_2_O_3_ at a temperature of 1200°C. Therefore, at the final step, the alumina powder was kept at 1200°C for 12 h to from *α*-Al_2_O_3_ completely before cooling down to room temperature in a desiccator. This material was denoted as *M*0 adsorbent.

### 2.3. Modification of *α*-Al_2_O_3_ by the SDS Adsorption

Prior to each modification experiment, the *α*-Al_2_O_3_ nanoparticles with the size range from about 30 to 40 nm (determined by TEM (transmission electron microscopy)) were vigorously mixed for 2 h by a multiple shaker. Then, the nanoparticles were sonicated for 20 min to eliminate particle aggregation. The *α*-Al_2_O_3_ adsorbents were modified by the addition of the appropriate volume of the stock SDS solution. All the adsorption experiments were carried out at pH 6 under the ionic strength condition of 0.01 M NaCl. All samples were thoroughly shaked for 2 h to reach the adsorption equilibrium.

### 2.4. Adsorptive Removal of RhB Using *α*-Al_2_O_3_ and SDS-Modified *α*-Al_2_O_3_

The SDS modified *α*-Al_2_O_3_ was washed with ultrapure water to remove the excess of SDS and to form *M*1 adsorbent. The RhB removal using synthesized *α*-Al_2_O_3_ (*M*0) and SDS modified *α*-Al_2_O_3_ (*M*1) was also carried out at room temperature (25 ± 2^o^C) under different conditions of pH, contact time, and adsorption dosage. Each adsorptive removal experiment was carried out at least three times. The RhB concentrations were quantified by ultraviolet visible (UV-Vis) spectroscopy at a wavelength of 554 nm using a spectrophotometer (UV-1650 PC, Shimadzu, Japan). The limit of detection (LOD) of UV-Vis spectroscopy for Rh determination was found to be 10^−8^ M. The removal (%) of RhB was determined by

(1) $$\begin{array}{c} \text{Removal}=\frac{C_{i}-C_{e}}{C_{i}}\times 100\%, \end{array}$$

where *C*_*i*_ and C_*e*_ are initial and equilibrium concentrations of RhB (mol/L), respectively.

The adsorption capacities of SDS onto *M*0 and RhB onto *M*1 were determined by

(2) $$\begin{array}{c} \Gamma =\frac{C_{i}-\;C_{e}}{m}\;\times M\times 1000, \end{array}$$

where Γ is the adsorption amount of RhB (mg/g), C_*i*_ the initial RhB concentration (mol/L), *C*_*e*_ is the equilibrium RhB concentration (mol/L), *M* is molecular weight of RhB (g/mol), and *m* is the adsorbent dosage (mg/mL).

The adsorption isotherms of RhB onto *M*1 were fitted by the two-step model with a general isotherm equation. The general isotherm equation [33] is

(3) $$\begin{array}{c} \Gamma =\frac{\Gamma_{\infty }k_{1}C\left(\left(1/n\right)+k_{2}C^{\text{n}-1}\right)}{1+k_{1}C\left(1+k_{2}C^{\text{n}-1}\right)}\text{, } \end{array}$$

where  Γ is the adsorption amount of RhB at concentration C, Γ_*∞*_ is the maximum adsorption capacity, *k*_1_ and *k*_2_ are equilibrium constants for in the first and second step, respectively, and *n* is cluster of adsorption. C is the equilibrium concentration of RhB.

To evaluate the adsorption mechanisms, the change in surface charge was evaluated by monitoring zeta (*ζ*) potential. The sample was added into a plastic capillary cell, then inserted in a laser velocimetry setup Zetasizer Nano ZS (Malvern Instruments, UK) under the electric field of 11.3 V/cm. Each measurement was repeated 3 times with 30 subruns. The *ζ* potential was calculated by Smoluchowski's equation [34]:

(4) $$\begin{array}{c} \zeta =\frac{u_{e}\;\eta }{\varepsilon_{rs}\;\varepsilon_{0}}, \end{array}$$

where *ζ* is the zeta potential (mV), *ս*_*e*_ is the electrophoretic mobility (µm cm/V.s), *η* is the dynamic viscosity of the liquid (mPa. s), *ɛ*_*rs*_ is the relative permittivity constant of the electrolyte solution (F/m), and *ɛ*_0_ is the electric permittivity of the vacuum (8.854 × 10^−12^ F/m).

## 3. Results and Discussion

### 3.1. Adsorption of SDS on the Synthesized *α*-Al_2_O_3_ Nanoparticles

**Figure 1:**
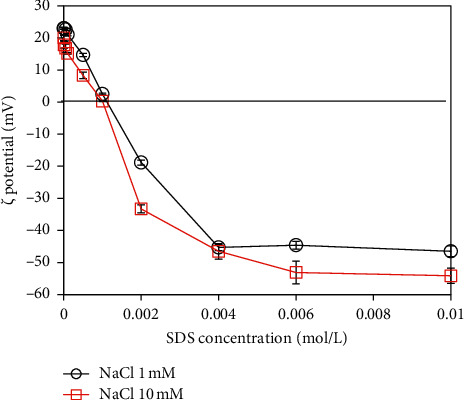
The *ζ* potential based on adsorption isotherms of the SDS onto *α*-Al_2_O_3_ nanoparticles at pH 5.0 and under different ionic strengths of 1 and 10 mM NaCl. The standard deviation was taken by the three different measurements.

The charging behavior of synthesized *α*-Al_2_O_3_ nanoparticles (*M*0) after modifying by SDS in the acid media (pH 5) and the different ionic strengths is represented in Figure 1. It can be seen that the charge sign of *M*0 changes and even reverses with the adsorption of anionic SDS. The tendency is consistent with the previous research studies in which the adsorption isotherm of the anionic SDS surfactants takes place into four regions or two steps [28, 35]. The zeta potential of M0 significantly decreases with the increment of SDS concentration, passing the isoelectric point (IEP), then moving to the saturated state in which the zeta potential keeps constant. In the first region of the low SDS concentration, the zeta potential of *M*0 decreases slowly until the neutral net charge due to the simple main electrostatic interactions between the anionic surfactants and oppositely charged alumina particles at pH 5. There is a sudden decrement of *ζ* potential shown in the second region due to the surfactant aggregation on the *α*-Al_2_O_3_ surface which is well-known as hemimicelles. The repulsions between SDS surfactants are shielded by the presence of electrolyte ions combining with the hydrocarbon chain forces, resulting in forming the SDS aggregates. In the third region, the surfactant aggregates continue to develop. In the last region, the zeta potential does not change beyond the critical micelle concentration (CMC).

The effect of ionic strength on the SDS adsorption onto *α*-Al_2_O_3_ nanoparticles is clarified. The *ζ* potential of *M*0 after adsorbing different concentrations of SDS was measured at pH 5 and under two ionic strength conditions. It is shown that at the fixed SDS concentration, the zeta potential of *α*-Al_2_O_3_ nanoparticles decreases with an increment of the ionic strength from 0.1 to 10 mM of NaCl. The SDS adsorption increased with increasing the NaCl concentration. The electrolyte ions shield not only the electrostatic forces between anionic SDS surfactants and oppositely charged alumina nanoparticles but also the repulsive forces between SDS surfactant molecules/or between the hemimicelles [36]. Herein, the later effect is stronger than the former one, resulting that more SDS surfactants adsorbed and formed the bilayer of admicelles on the *M*0 surface [31, 37]. Therefore, the surface charge of *α*-Al_2_O_3_ remained the highly negatively charged that is useful to remove cationic dye RhB. The use of 0.006 M SDS at 10 mM NaCl is suitable to form SDS-modified *α*-Al_2_O_3_ (*M*1) material.

### 3.2. Adsorptive Removal of RhB Using Different Adsorbents

#### 3.2.1. Effect of pH

**Figure 2:**
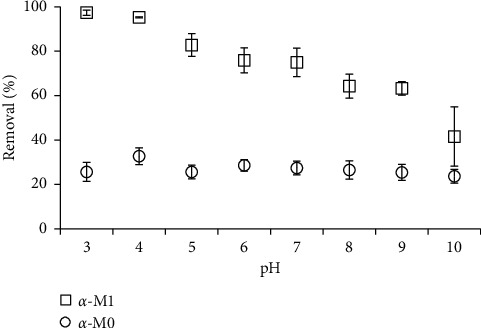
The effect of pH on RhB removal using *α*-*M*0 and *α*-*M*1 materials at 1 mM NaCl (C_*i*_ (RhB) = 10^−5^ M, adsorbent dosage of 5 mg/mL, and contact time of 30 min).

The pH solution is one of the most important parameter influences to RhB removal using *M*0 and *M*1 materials. The pH solution strongly influences to the surface charge of adsorbent *M*0 and the desorption of SDS for modified adsorbent *M*1 [31, 38]. The influence of pH on RhB removal using *M*0 and *M*1 was carried out from pH 3 to 10 at 1 mM NaCl using adsorbent dosage of 5 mg/mL, with a contact time of 30 min (Figure 2).

Figure 2 shows that the RhB removal using *M*0 material did not change significantly except for pH 4 in pH range of 3–10 while the RhB removal reduced with an increase of pH from 3 to 10 when using *M*1 material. At pH 3, *α*-Al_2_O_3_ may dissolve into solution so that the error bars show the standard deviations were high for *M*1 [39]. It should be noted that the *M*0 surface charge decreases with increasing pH but the net charge density of *M*0 is small. Since RhB is positive charged in the pH range so that RhB removal using *M*0 was rather low (around 25%). For *M*1 material, the SDS desorption enhanced with increasing pH so that the net negative charge of *M*1 decreased [38]. In all pH ranges, the RhB removal using *M*1 was much higher than that using *M*0 under the same experimental conditions. Figure 2 also indicates that the RhB removal achieved 95.2 and 32.7% at pH 4 when using *M*1 and *M*0 materials, respectively. Thus, we kept pH 4 for further investigation on RhB removal using both *M*0 and *M*1 materials.

#### 3.2.2. Effect of Adsorbent Dosage

**Figure 3:**
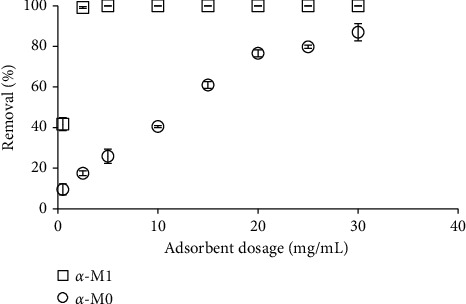
The effect of adsorbent dosage on RhB removal using *α*-*M*0 and *α*-*M*1 materials at 1 mM NaCl (C_*i*_ (RhB) = 10^−5^ M, pH 4, and contact time of 30 min).

For adsorption technique, binding site and specific surface area highly affect the removal efficiency because they can change the surface charge density of adsorbent [40]. The amounts of *M*0 and *M*1 materials were changed from 0.5 to 30 mg/mL (Figure 3). Figure 3 shows that the RhB removal using *M*0 and *M*1 materials increased with increasing adsorbent dosage but the RhB removal using *M*1 achieved the highest efficiency with very small amount of adsorbent. The adsorbent dosage 5 mg/mL is suitable to get the removal approximately 100% for *M*1 while the RhB removal using *M*0 was only 26% with such adsorbent dosage. Thus, optimal adsorbent dosage for *M*1 was 5 mg/mL.

Because the RhB removal using *M*1 was extremely higher that using *M*0, further studies only investigate on the adsorption of RhB onto M1 material.

#### 3.2.3. Effect of Contact Time

**Figure 4:**
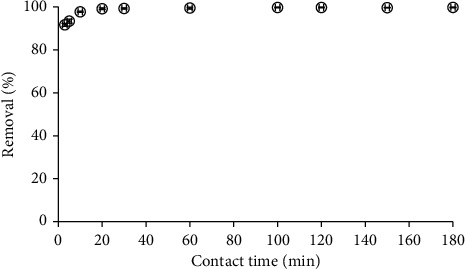
The effect of contact time on RhB removal using *M*1 material (C_*i*_ (RhB) = 10^−5^ M, pH 4, and adsorbent dosage 5 mg/mL).

Contact time is known as the time from initial mixing RhB with adsorbent. The RhB removal using *M*1 in the contact time range of 0–180 min is shown in Figure 4.

Figure 4 shows that the contact time for RhB removal using *M*1 reached the equilibrium with only 30 min. This time is much faster than RhB adsorption on well-known adsorbent as activated carbon (120 min) [41]. Therefore, contact time of 30 min was fixed for RhB removal using *M*1 material.

### 3.3. Adsorption Mechanisms of RhB onto Synthesized **α**-Al_2_O_3_ Nanoparticles with SDS Modification (*M*1)

**Figure 5:**
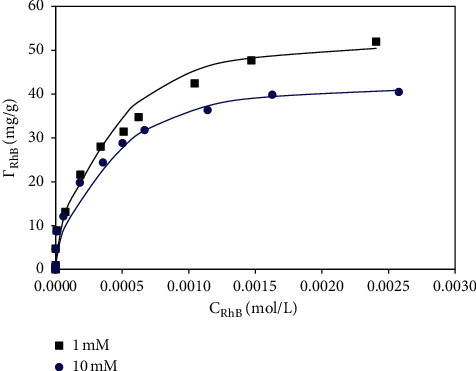
Adsorption isotherms of RhB onto SDS-modified *α*-Al_2_O_3_ nanoparticles (*M*1) at two NaCl concentrations. The points are experimental data while solid lines are fitted by the two-step adsorption model.

Adsorption isotherms are important to understand adsorption mechanisms of RhB onto synthesized *α*-Al_2_O_3_ nanoparticles with SDS modification (*M*1).  Figure 5 shows that at acid media (pH 4), the adsorption of RhB at low ionic strength was always higher than at high ionic strength. At high NaCl concentration, the total counter ions are high that can screen the surface charge of *M*1. As a result, the net negative charge of *M*1 increased (see Figure 1) while the electrostatic attraction between cationic RhB and negatively charged *M*1 surface was decreased. We suggest that RhB adsorption onto *M*1 is mainly controlled by electrostatic attraction and effect of ionic strength on RhB adsorption is important.

As can be seen in Figure 5, the data point represented that experimental results of RhB adsorption onto *M*1 were in accordance with a two-step adsorption model using the fit parameters in Table 1. Table 1 and Figure 5 show that the plateau RhB adsorption at 1 mM NaCl was higher than at 10 mM. Interestingly, the same fit parameters of *k*_2_ and *n* could be used for two isothermal adsorptions at 1 and 10 mM NaCl. Nevertheless, the *k*_1_ at 1 mM NaCl was slightly higher than *k*_1_*t* at 10 mM. It implies that the *k*_1_ can be useful to predict the electrostatic interaction of RhB adsorption onto SDS-modified *α*-Al_2_O_3_ nanoparticles. The maximum adsorption capacity of RhB using *M*1 materials was found to be 52 mg/g that was much higher than many reported adsorbents [42].

**Figure 6:**
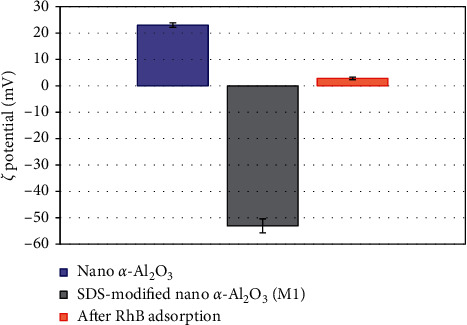
The *ζ* potential of synthesized nano *α*-Al_2_O_3_, SDS-modified nano *α*-Al_2_O_3_ (*M*1), and *M*1 after RhB adsorption in 10 mM NaCl (pH 5).

To confirm the adsorption mechanism, the charging behavior of *α*-Al_2_O_3_ nanoparticles before and after adsorption was considered.  Figure 6 indicates that the *ζ* potential of synthesized *α*-Al_2_O_3_ was about +23.0 mV at pH 5. The charge reversal was taken place after SDS adsorption so that a negative charge of *M*1 was achieved (*ζ* = - 53.1 mV). Due to the presence of admicelles with local bilayer onto *α*-Al_2_O_3_ surface, the surface charge of *α*-Al_2_O_3_ was highly negative. [31, 38]. However, after RhB adsorption, a small positive of *ζ* was obtained. The change of *ζ* potential indicates that RhB adsorption onto *M*1 material was controlled by electrostatic interaction that agrees well with the results of adsorption isotherms. In other word, we can demonstrate that electrostatic is the main driving force that induces RhB adsorption onto SDS-modified *α*-Al_2_O_3_ nanoparticles.

### 3.4. The Reuse Potential and the Application of SDS-Modified Nano *α* -Al_2_O_3_

**Figure 7:**
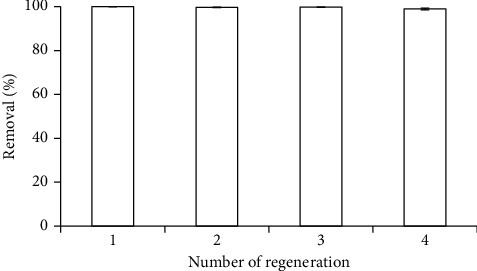
Removal of RhB using *M*1 after four regenerations. Error bars show standard deviation of three replicates.

The reuse potential of material is needed to examine the stability and regeneration of *M*1 adsorbent. The *M*1 absorbent was regenerated by using 0.1 M NaOH.  Figure 7 shows the RhB removal after regeneration cycles. It is clear to observe that the RhB removal changed insignificantly after four regenerations. The RhB removal was still higher than 98%, indicating that *M*1 adsorbent-based SDS-modified nano *α* -Al_2_O_3_ was highly reusable and high performance of RhB removal.

The application *M*1 adsorbent in wastewater samples is important to evaluate the performance of adsorbent. The wastewater samples of a textile company in the Pho Noi industrial zone in Hung Yen Province, Vietnam, were collected at three different discharged locations and analyzed in the same day. Then, the textile samples were centrifuged to remove the solids and the solutions were collected. RhB in each textile wastewater sample was removed under optimal conditions.  Table 2 shows the RhB removal from three wastewater samples using *M*1 adsorbent. Although the RhB removal is strongly influenced by many factors in actual samples, the RhB removal in all samples reached about 100%. Our results again indicate that SDS-modified nano *α*-Al_2_O_3_ is a high-performance adsorbent for the cationic dye removal from wastewater.

## 4. Conclusions

We have reported a scientific research on the RhB removal using synthesized *α*-Al_2_O_3_ nanomaterial with surface modification by anionic surfactant SDS. The RhB removal using SDS-modified *α*-Al_2_O_3_ was much higher than raw *α*-Al_2_O_3_. The suitable parameters for RhB removal using SDS-modified *α*-Al_2_O_3_ were contact time 30 min, pH 4, and adsorbent dosage 5 mg/mL. The maximum adsorption capacity of RhB was found to be 52.0 mg/g while the removal reached 100%. Adsorption isotherms of RhB onto SDS-modified *α*-Al_2_O_3_ were in accordance with the two-step adsorption model. Based on the change in surface charge monitoring by zeta potential and adsorption isotherm, we indicate that electrostatic attraction was the main driving force induced adsorption. The SDS-modified *α*-Al_2_O_3_ is reusable adsorbent for RhB removal with very high efficiency of greater than 98% after four regeneration cycles while the RhB removal using the this adsorbent reached to about 100% for actual textile wastewater.

## Figures and Tables

**Table 1:** The fit parameters for adsorption RhB onto SDS-modified *α*-Al_2_O_3_ (*M*1).

*C*_NaCl_ (mM)	Γ (mg/g)	*k*_1_ (10^3^ g/mg)	*k*_2_ (10^3^ g/mg)^n−1^	n
10	42	25	2000	2.9
1	52	30	2000	2.9

**Table 2:** The removal of RhB from textile wastewater using SDS-modified nano *α* -Al_2_O_3_.

Wastewater sample	RhB concentration before treatment (×10^6^ M)	RhB concentration after treatment (M)	Removal (%)
S1	5.7	<LOD	∼100
S2	10.5	<LOD	∼100
S3	10.6	<LOD	∼100

## Data Availability

The data and supporting materials are included within the article.
